# Role of Maspin, CK17 and Ki-67 Immunophenotyping in Diagnosing of Pancreatic Ductal Adenocarcinoma in Endoscopic Ultrasound-Guided Fine Needle Aspiration Cytology

**DOI:** 10.31557/APJCP.2021.22.10.3299

**Published:** 2021-10

**Authors:** Mona M Mamdouh, Hussein Okasha, Hebat Allah M Shaaban, Nesreen H Hafez, Emad Hamza El-Gemeie

**Affiliations:** 1 *Department of Pathology, National Cancer Institute, Cairo University, Cairo, Egypt. *; 2 *Department of Internal Medicine, Gastroenterology and Hepatology division, Kasr Al- Aini Hospitals, Cairo University, Cairo, Egypt. *

**Keywords:** Pancreatic ductal adenocarcinoma, EUS-FNAC, Maspin, CK17, Ki-67

## Abstract

**Background and study aim:**

One of the problems in diagnosing pancreatic ductal adenocarcinoma (PDAC) is differentiation between PDAC cells and benign pancreatic tissue cells in cytologic samples. This study aimed to evaluate the usefulness of Maspin, CK17 and Ki-67 immunocytochemistry (ICC) in differentiation between these two groups of cells.

**Materials and methods:**

This retrospective study was carried on 80 cases of PDAC and 25 cell blocks of benign pancreatic tissue cells as a control group for evaluation of Maspin, CK17 and Ki-67 ICC. PDAC cases were sampled by endoscopic ultrasound guided fine needle aspiration cytology (EUS-FNAC), while cell blocks of control group were aspirated from benign pancreatic tissues that were obtained from the pancreatic surgically resected specimens. Immunostaining patterns, sensitivity, specificity, positive predictive value (PPV), negative predictive value (NPV) and accuracy of each antibody as well as possible antibody combined panels of these markers in differentiation between the two groups were evaluated.

**Results:**

Positive immunoreactivity for Maspin, CK17 and Ki-67 were 92.5%, 80% and 72.5% in PDAC cases, respectively. In contrast to PDAC cases, all the cell blocks of benign pancreatic tissue cells were negative for these markers. Regarding different panels, combined use of Maspin, CK17 and Ki-67 together as a triple test (at least one of them is positive) achieved the highest sensitivity of 98.8%, specificity of 100%, PPV of 100%, NPV of 96.2% and accuracy of 99% in the differentiation between PDAC and benign pancreatic tissue.

**Conclusion:**

Employing this short panel [Maspin, CK17 and Ki-67] is helpful for better differentiation between PDAC and benign pancreatic tissue.

## Introduction

Pancreatic ductal adenocarcinoma (PDAC) accounts for nearly 85% of solid pancreatic tumors (Reid and Centeno, 2014; Aksoy-Altinboga et al., 2018). PDAC is the fourth leading cause of cancer-related death in the world (Foucher et al., 2018). Approximately 80% of patients with pancreatic cancer have unresectable disease at diagnosis because of early loco-regional extension or distant metastases (He et al., 2014). Recent studies reported the potentiality of neoadjuvant therapy to decrease the size of tumors and make them more resectable, therefore a definite preoperative diagnosis of PDAC is important for all patients (Furuhata et al., 2017). Endoscopic ultrasound (EUS) is one of the best methods for detecting pancreatic cancer (Dimastromatteo et al., 2017). In addition, endoscopic ultrasound-guided fine needle aspiration cytology (EUS-FNAC) for pancreatic lesions is a safe and efficient procedure and has become a popular approach for obtaining diagnostic pancreatic samples (Kudo et al., 2014).

The EUS-FNAC is a well-established method to accurately diagnose high-grade PDAC, while interpretation of low grade PDAC can be challenging to be differentiated from benign pancreatic tissue (Dim et al., 2014; Furuhata et al., 2017), even in tissue biopsies (Liu et al., 2012). Application of appropriate immunocytochemistry (ICC) markers on cell blocks enables cytopathologists to differentiate PDAC from benign mimickers (Lin et al., 2015; Furuhata et al., 2017). A growing number of potential immunostaining markers in diagnosing PDAC have emerged, however reports that have specifically focused on distinguishing PDAC from benign mimickers, especially in FNAC, are very limited (Furuhata et al., 2017). Some studies showed that expression of Maspin (Cao et al., 2007; Liu et al., 2012; Berardi et al., 2013; Lok et al., 2014; Furuhata et al., 2017; Aksoy-Altinboga et al,. 2018), CK17 (Goldstein and Bassi, 2001; Chu et al., 2005; Sarbia et al., 2007; Yang et al., 2012; Berardi et al., 2013) and Ki-67 (Klein et al., 2002; Jahng et al., 2010; Karamitopoulou et al., 2010; Kim et al., 2015) are conspicuous for diagnosing PDAC.

Maspin is a unique member of the serpin superfamily of serine proteinase inhibitors and is located on chromosome 18q21.3-q23. Maspin was originally described as a tumor suppressor gene inhibiting cell motility, invasiveness and metastases (Umekita et al., 2006). However, the correlation between Maspin immunostaining expression and worse prognoses was reported in many cancers (Umekita et al., 2002; Hirai et al., 2005). Maspin was documented to be overexpressed in PDAC in both tissue biopsies and cell blocks obtained by FNAC (Cao et al., 2007; Liu et al., 2012; Berardi et al., 2013; Lok et al., 2014; Furuhata et al., 2017; Aksoy-Altinboga et al., 2018).

CK17 is a low-molecular-weight keratin that is normally expressed in myoepithelial, basal cells and subsets of hair shaft epithelia (Chu et al., 2005; Lok et al., 2014). It is also expressed mainly in squamous, basal and transitional cell carcinomas as well as in adenocarcinomas with squamous differentiation (Goldstein and Bassi, 2001; Yang et al., 2012). Few studies had shown that CK17 also might be a useful marker for the diagnosis of pancreaticobiliary adenocarcinomas (Goldstein and Bassi, 2001; Berardi et al., 2013) and separating them from extra-pancreaticobiliary non-mucinous adenocarcinomas (Chu et al., 2005; Sarbia et al., 2007; Yang et al., 2012).

Ki-67 is a nuclear protein that is increased in proliferating cells and present in all phases of the cell cycle, except the resting phase (Jahng et al., 2010). Overexpression is frequently seen in a variety of malignant tumors and is associated with worse prognosis (Stuart-Harris et al., 2008; Viale et al., 2008). A step-wise progression from normal pancreatic epithelium to pancreatic intraepithelial neoplasia (PanIN) and then to frank adenocarcinoma has been correlated with increasing Ki-67 labeling index expression (Klein et al., 2002; Jahng et al., 2010; Karamitopoulou et al., 2010; Kim et al., 2005; Furuhata et al., 2017).

Therefore, this study aimed to evaluate the usefulness of Maspin, CK17 and Ki-67 ICC as separate markers and as different combined panels in differentiation between PDAC cells and benign pancreatic tissue cells.

## Materials and Methods

This retrospective study was conducted on 80 cases of PDAC and 25 cell blocks of benign pancreatic tissue cells as a control group in National Cancer Institute (NCI), Cairo University.

*EUS-FNAC procedure and preparation of the cases*

The aforementioned 80 cases were aspirated by EUS-FNAC that was performed using 22-gauge needle with rapid-on-site evaluation (ROSE) in the Endoscopic Unit. Smears were prepared from the aspirate of the needle pass: at least, one was air-dried and Diff-Quik-stained (used for ROSE) and the other was fixed in 95% Ethyl alcohol for minutes at room temperature. If the obtained sample was non-diagnostic, additional passes were attempted until diagnostic material is obtained. Additional passes or needle rinse material was collected in solution [10% neutral-buffered formalin: alcohol, 1:9] for subsequent preparation of cell block. In addition, some material was collected in SurePath liquid based cytology (LBC) preservative. 

All the aspirated material was sent to the Cytology Unit, for preparation, immunostaining and cytological diagnosis. The Ethyl alcohol-fixed slides were stained using Papanicolaou stain, while cell block material was prepared and stained with HandE stain. Whereas Surepath LBC material was prepared and stained using SurePath LBC apparatus.

Regarding the 25 cell blocks of control group, they were prepared from aspirated benign pancreatic tissues that were obtained from the pancreatic surgically resected specimens that were sent to our Pathology Unit (25 Whipple operations for PDAC) and then stained using HandE stain.

*Selection of cases*

These PDAC cases were retrieved from the database of Cytology Unit, during the period from January 2014 to December 2019. The selected criteria of the cases included unequivocal cytologic reports of PDAC, availability of all studied correlated clinical and radiologic/endoscopic data, availability of formalin-fixed paraffin-embedded cell blocks with adequate tumor cells to perform ICC and no administered chemo- or radiotherapy before FNAC. 

*Cytologic examination and interpretation*

The review of diagnosis and grading of our cases was done according to “The Papanicolaou Society of Cytopathology System for Reporting Pancreaticobiliary Cytology” (Pitman et al., 2014). The diagnosis of the cases was correlated with their final histopathological diagnoses (if present) and radiologic/endoscopic data that included malignant criteria of the pancreatic mass, pathological lymph nodes and/or presence of distant metastases. Also clinical course was considered.

*Immunocytochemical (ICC) staining*

For immunostaining, Paraffin embedded sections of cell blocks of PDAC cases and the control group were made at 4 microns thickness and mounted on positive charged slides. Immunostaining was done by BenchMark IHC/ISH staining module (Ventana immunostaining system), the following steps occurred automatically: 1) Deparaffinization, 2) Cell conditioning (Standard CC1 application) for 80 minutes, 3) Application of one drop (100µ) of the antibody, 4) Application of cover slip and incubation for 32 minutes, 5) Application of one drop of DAB (counterstain) with Haematoxylin II and incubation for 8 minutes, 6) Application of Bluing Reagent for 4 minutes, 7) Slides were extracted and arranged in racks, 8) Slides were washed in tap water and soap for 5 minutes and then dehydrated in the ascending grades of alcohol for 5 minutes in each container, 9) Slides were cleared in xyline, and then cover slips were applied.

Appropriate positive and negative controls were included in each run. The selected markers were Maspin (clone BSB-92, Ready to use, Gene Tex), CK17 (clone E3, Ready to use, Dako) and Ki-67 (clone MIB-1, Ready to use, Dako).

*Immunocytochemical evaluation, scoring and interpretation*

ICC evaluation was done, including assessment of subcellular immunostaining localization, the percentage of positively stained cells and the immunostaining intensity. Nuclear and/or cytoplasmic immunostaining for Maspin, cytoplasmic immunostaining for CK17 and nuclear immunostaining for Ki-67 were considered as a definite positivity. The percentage of immunostained tumor cells was recorded as negative (<5% of tumor cells stained), 1+ (5%–25%), 2+ (26%–50%), 3+ (51%–75%), and 4+ (>75%), hence the cutoff point of positivity for all studied markers is 5% (Chu et al., 2005; Aksoy-Altinboga et al., 2018). Scores 1+ and 2+ were considered “focal immunostaining”, while scores 3+ and 4+ were considered “diffuse immunostaining” (Chu et al., 2005; Cao et al., 2007; Liu et al., 2012; Lok et al., 2014). The immunostaining intensity was graded as: no immunostaining, weak immunostaining, moderate immunostaining and strong immunostaining (Goldstein and Bassi, 2001; Karamitopoulou et al., 2010; Yang et al., 2012; Lok et al., 2014). 

We correlated immunostaining results of PDAC cases with some clinico-pathological data.

*Statistical analysis*

Data management and analysis were performed using Statistical Package for Social Sciences (SPSS) vs. 25. Numerical data were summarized using means and standard deviations or medians and/or ranges, as appropriate. Categorical data were summarized as numbers and percentages. Numerical data were explored for normality using Kolmogrov-Smirnov test and Shapiro-Wilk test. Chi square or Fisher’s tests was used to compare between independent groups with respect to categorical data. Comparisons between two groups for normally distributed numeric variables were done using the Student’s t-test while for non-normally distributed numeric variables, comparisons were done by Mann-Whitney U test. To measure the strength of association between the non-normally distributed measurements, Spearman’s correlation coefficients was calculated (r is the correlation coefficient, it ranges from -1 to +1), (+1 indicates positive correlation, -1 indicates negative correlation, 0 indicates no correlation). Receiver operating characteristic curve (ROC curve) was done to determine the best sensitivity, specificity and area under the curve. The accuracy of the test depends on how well the test separates the group being tested into those with and without the disease in question. All tests were two tailed and Probability (p-value) ≤ 0.05 is considered significant.

## Results

Our PDAC cases included 51 male and 29 female with male: female ratio of 1.8:1 and a mean age of 60 years. Among the studied cases, recorded reports of CA19-9 serum level were only available for 63 cases and those of CEA serum level were available for 58 cases. High CA19-9 and CEA serum levels were documented in 74.6% and 37.9% of available reports, respectively. The majority of our cases were located at the head of the pancreas, represented 71.2%. Regarding TNM staging groups, the majority of our cases were of high stage; stage III and IV represented 15% and 43.8%, respectively. Most of cases were moderately differentiated tumors, represented 63.7% (Table 1).

*Immunocytochemical findings*

The immunostaining results for PDAC cases are summarized in (Table 2). Out of the studied 80 PDAC cases, 74 (92.5%) were positive for Maspin, with the majority showed strong intensity, represented 90.54%. Among Maspin positive PDAC cases, 57 cases (77%) showed both nuclear and cytoplasmic immunostaining, while 16 cases (21.6%) showed nuclear immunostaining and only 1 case (1.4%) showed cytoplasmic immunostaining. CK17 was positive in 64 out of 80 PDAC cases (80%), with strong intensity in the majority of positive cases, represented 90.63%. Ki-67 expression was noted in 58 (72.5%) out of 80 studied PDAC cases. Regarding Ki-67 immunostaining intensity, the majority of positive PDAC cases showed strong intensity, represented 79.31%. The majority of positive PDAC cases for Maspin and CK17 exhibited diffuse immunostaining, represented 70.27% and 59.37%, respectively, whereas Ki-67 showed focal immnunostaining in 77.59% of the cases (Figure 2 and 3).

In contrast to PDAC cases, all the cell blocks of benign pancreatic tissue cells were negative for Maspin, CK17 and Ki-67 (Figure 4), with statistical significant difference in differentiation between the two groups (P < 0.01).

In differentiation of PDAC from benign pancreatic tissue, Maspin showed sensitivity of 92.5%, NPV of 80.6% and accuracy of 94.2%. CK17 sensitivity, NPV and accuracy were of 80%, 61% and 84.8%, respectively. Finally, Ki-67 had sensitivity of 72.5%, NPV of 53.2% and accuracy of 79%. Specificity and PPV were 100% for each marker. Regarding different panels, combined use of the three markers together as a triple test (at least one of them is positive) achieved the highest sensitivity of 98.8 %, NPV of 96.2 % and accuracy of 99 % in the differentiation between PDAC and benign pancreatic tissue. Whereas, specificity and PPV were 100% for each of the studied panels (Table 3).

Among all studied clinico-pathological data, Ki-67-positive PDAC cases only showed statistical significant relation with tumor grade, compared with negative ones. Ki-67 positivity was significantly higher in poorly differentiated PDAC, represented 22 out of 24 cases (91.7%), compared to well and moderately differentiated tumors, represented 3 out of 5 cases (60%) and 33 out of 51 cases (64.7%), respectively. In contrast, both Maspin and CK17 immunostaining were not significantly correlated with all studied clinico-pathological data (Table 4).

**Figure 1:**
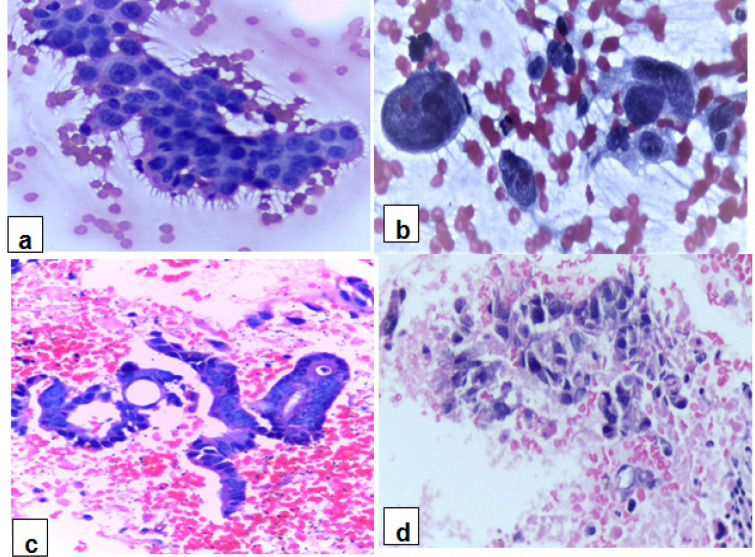
(a&b) cytologic smears of PDAC, showing nuclear enlargement, nuclear membrane irregularity and anisonucleosis: (a) Malignant ductal epithelial cells, arranged in an uneven, “drunken honeycomb” sheet (Papanicolaou-stained x40) and (b) loosely cohesive syncytial tissue fragments, with some separate forms (Papanicolaou-stained x40). (c and d) cell block sections of PDAC: (c) Irregular and angulated tubules and (d) loosely cohesive sheets (H&E-stained ×40)

**Table 1:** Clinico-Pathological Data of Our 80 Cases of PDAC

Criterion		Variables	n (%)
Sex (n=80)		Female	29 (36.2)
		Male	51 (63.8)
Age (n=80)		Mean ±SD	60±10
		Median (range)	59 (28-77)
Serum markers*			
	CA19-9 (n=63)	High	47 (74.6)
		Normal	16 (25.4)
	CEA (n=58)	High	22 (37.9)
		Normal	36 (62.1)
Site (n=80)		Head	57 (71.2)
		Body	6 (7.5)
		Head & body	5 (6.3)
		Body & tail	5 (6.3)
		Tail	7 (8.7)
TNM staging**			
	T-stage	T1	2 (2.5)
		T2	7 (8.7)
		T3	46 (57.5)
		T4	25 (31.3)
	N-stage	N0	27 (33.7)
		N1	53 (66.3)
	M-stage	M0	45 (56.2)
		M1	35 (43.8)
	Staging group	I	2 (2.5)
		IIA	8 (10)
		IIB	23 (28.7)
		III	12 (15)
		IV	35 (43.8)
Grade		Well	5 (6.3)
		Moderate	51 (63.7)
		Poorly differentiated	24 (30)

*, NCI normal references for CA19-9 & CEA; 0-22.3 U/ml and 0-5 ng/ml, respectively; **, TNM staging according to AJCC/UICC (Cong et al., 2018)

**Table 2:** Immunocytochemistry (ICC) Findings for Maspin, CK17 and Ki-67 of our 80 Cases of PDAC

Marker	Positive cases	Percentage of stained tumor cells				Immunostaining intensity		
		1	2	3	4	weak	moderate	strong
	n (%)	n (%)	n (%)	n (%)	n (%)	n (%)	n (%)	n (%)
Maspin	74 (92.5)	8 (10.81)	14 (18.92)	16 (21.62)	36 (48.65)	3 (4.05)	4 (5.41)	67 (90.54)
CK17	64 (80)	20 (31.25)	6 (9.38)	17 (26.56)	21 (32.81)	2 (3.12)	4 (6.25)	58 (90.63)
Ki-67	58 (72.5)	31 (53.45)	14 (24.14)	9 (15.51)	4 (6.90)	3 (5.17)	9 (15.52)	46 (79.31)

**Table 3:** The Sensitivity, Specificity, PPV, NPV and Accuracy of Maspin, CK17 and Ki-67 ImmunocytoChemistry (ICC), each one Individually and as Different Panels

ICC markers	Sensitivity	Specificity	PPV	NPV	Accuracy
	(%)	(%)	(%)	(%)	(%)
Maspin	92.5	100	100	80.6	94.2
CK17	80	100	100	61	84.8
Ki-67	72.5	100	100	53.2	79
Maspin/CK17/Ki-67 panel*	98.8	100	100	96.2	99
Maspin/CK17 panel*	97.5	100	100	92.6	98.1
Maspin/Ki-67 panel*	95	100	100	86.2	96.2
CK17/Ki-67 panel*	92.5	100	100	80.6	94.3

PPV, Positive Predictive Value ; NPV, Negative Predictive Value; *, The test was considered positive if any of the markers was positive.

**Figure 2:**
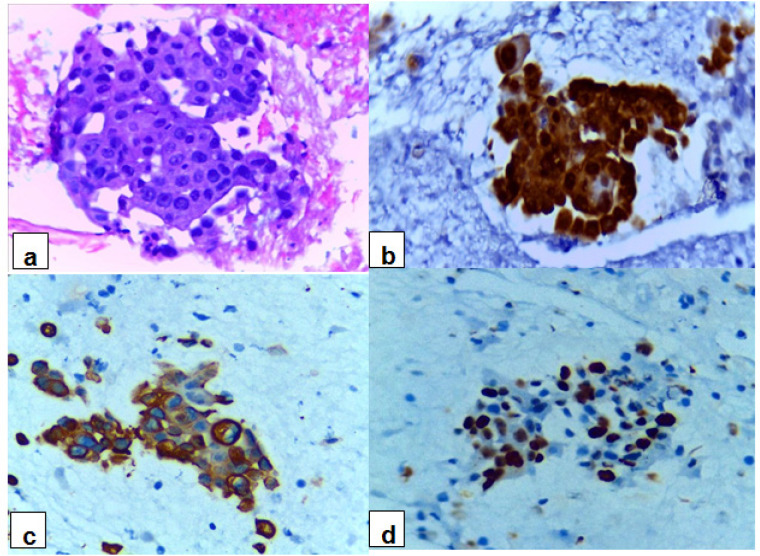
A Case of PDAC Reveals Malignant Pancreatic Ductal Cells: (a) cell block section (H&E-stained x40), (b) Maspin shows diffuse positive nuclear and cytoplasmic immunostaining for malignant cells with strong intensity (x40), (c) CK17 shows diffuse positive cytoplasmic immunostaining for malignant cells with strong intensity (x40) and (d) Ki-67 shows diffuse positive nuclear immunostaining for malignant cells with strong intensity (x40).

**Figure 3:**
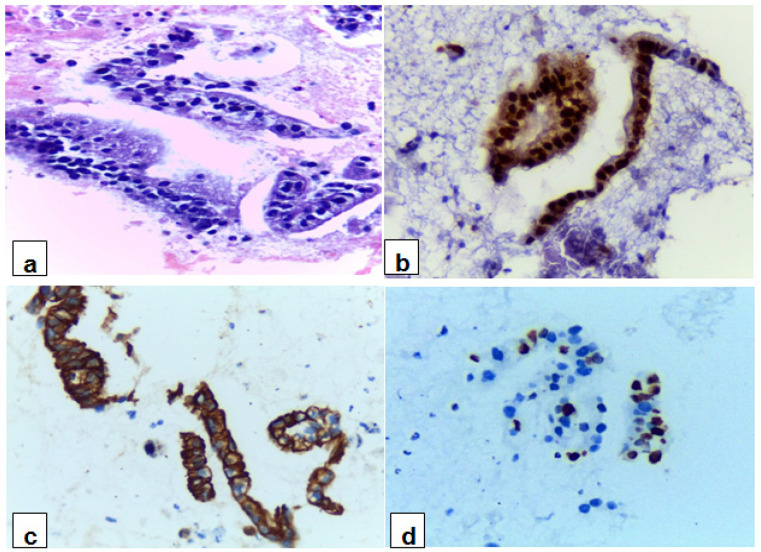
A cases of PDAC Reveals Malignant Pancreatic Ductal Cells: (a) cell block section (H&E-stained x40), (b) Maspin shows diffuse positive nuclear and cytoplasmic immunostaining for malignant cells with strong intensity (x40), (c) CK17 shows diffuse positive cytoplasmic immunostaining for malignant cells with strong intensity (x40) and (d) Ki-67 shows focal positive nuclear immunostaining for malignant cells with strong intensity (x40).

**Table 4:** Relationship between Clinico-Pathological Data of PDAC Cases and Studied Markers

Clinico-pathologic variables		Mapsin			CK17			Ki67		
		Positive	Negative	p	Positive	Negative	p	Positive	Negative	p
		n (%)	n (%)		n (%)	n (%)		n (%)	n (%)	
Age (n=80)										
	Mean ±SD	60 ±10	61 ±8	0.862	61±10	56±9	0.116	60±9	59±11	0.592
	Median (range)	59 (28-77)	58 (54-73)		61 (28-77)	56 (36-72)		60 (43-75)	59 (28-77)	
Sex (n= 80)										
	Female	28 (96.6)	1 (3.4)	0.409	24 (82.8)	5 (17.2)	0.775	25 (86.2)	4 (13.8)	0.067
	Male	46 (90.2)	5 (9.8)		40 (78.4)	11 (21.6)		33 (64.7)	18 (35.3)	
CA19-9 (n= 63)										
	High	43 (91.5)	4 (8.5)	1	40 (85.1)	7 (14.9)	0.448	32 (68.1)	15 (31.9)	0.757
	Normal	15 (93.8)	1 (6.2)		12 (75)	4 (25)		12 (75)	4 (25)	
CEA (n= 58)										
	High	20 (90.9)	2 (9.1)	0.63	20 (90.9)	2 (9.1)	0.088	15 (68.2)	7 (31.8)	0.35
	Normal	34 (94.4)	2 (5.6)		26 (72.2)	10 (27.8)		29 (80.6)	7 (19.4)	
Anatomical site (n= 80)										
	Head	51 (89.5)	6 (10.5)	NA	45 (78.9)	12 (21.1)	NA	43 (75.4)	14 (24.6)	NA
	Body	6 (100)	0 (0)		6 (100)	0 (0)		2 (33.3)	4 (66.7)	
	Head& body	5 (100)	0 (0)		4 (80)	1 (20)		5 (100)	0 (0)	
	Body& tail	5 (100)	0 (0)		4 (80)	1 (20)		3 (60)	2 (40)	
	Tail	7 (100)	0 (0)		5 (71.4)	2 (28.6)		5 (71.4)	2 (28.6)	
T stage (n= 80)										
	T1	2 (100)	0 (0)	NA	2 (100)	0 (0)	NA	2 (100)	0 (0)	NA
	T2	7 (100)	0 (0)		6 (85.7)	1 (14.3)		5 (71.4)	2 (28.6)	
	T3	41 (89.1)	5 (10.9)		33 (71.7)	13 (28.3)		31 (67.4)	15 (32.6)	
	T4	24 (96)	1 (4)		23 (92)	2 (8)		20 (80)	5 (20)	
N stage (n= 80)										
	N0	25 (92.6)	2 (7.4)	1	22 (81.5)	5 (18.5)	0.813	20 (74.1)	7 (25.9)	0.822
	N1	49 (92.5)	4 (7.5)		42 (79.2)	11 (20.8)		38 (71.7)	15 (28.3)	
M stage (n= 80)										
	M0	41 (91.1)	4 (8.9)	0.691	35 (77.8)	10 (22.2)	0.779	32 (71.1)	13 (28.9)	0.805
	M1	33 (94.3)	2 (5.7)		29 (82.9)	6 (17.1)		26 (74.3)	9 (25.7)	
Staging group (n=80)										
	Low (1A, IIA, IIB)	30 (90.9)	3 (9.1)	0.687	24 (72.7)	9 (27.3)	0.256	22 (66.7)	11 (33.3)	0.446
	High (III, IV)	44 (93.6)	3 (6.4)		40 (85.1)	7 (14.9)		36 (76.6)	11 (23.4)	
Grade (n= 80)										
	Well	5 (100)	0 (0)	NA	5 (100)	0 (0)	0.729	3 (60)	2 (40)	0.041
	Moderate	47 (92.2)	4 (7.8)		40 (78.4)	11 (21.6)		33 (64.7)	18 (35.3)	
	High	22 (91.7)	2 (8.3)		19 (79.2)	5 (20.8)		22 (91.7)	2 (8.3)	

**Figure 4:**
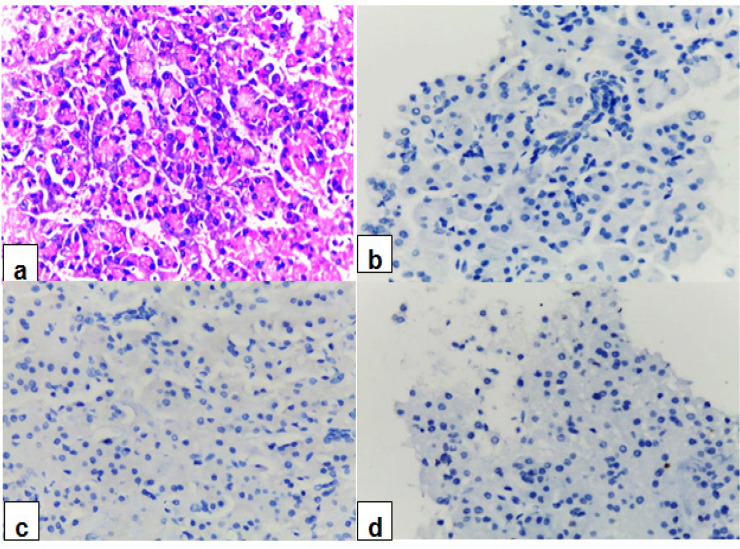
Normal Pancreatic Tissue (Control Group), Aspirated from the Pancreatic Surgically Resected Specimens: (a) H&E-stained cell block section (x40), (b) Maspin shows negative immunostaining (x40), (c) CK17 shows negative immunostaining (x40) and (d) Ki-67 shows negative immunostaining (x40)

## Discussion

Differentiation of PDAC, especially low grades, from benign pancreatic tissues is one of the obstacles that facing cytopathologists in the diagnosis of pancreatic lesions in EUS-FNAC (Furhata et al., 2017). In this study we aimed to providing information about a new combination of ICC antibodies (Maspin, CK17 and Ki-67) as an ancillary study in diagnosis of EUS-FNAC of PDAC. Although many studies evaluated one or two of these markers in diagnosis of PDAC (Yang et al., 2012; Furuhata et al., 2017; Aksoy-Altinboga et al., 2018), to our best knowledge, we are the first in the literature to test the combination of these three markers together and we are also the first to study the usefulness of CK17 in diagnosing PDAC on cytologic material.

In our study, we found that Maspin, CK17 and Ki-67 were very useful markers in differentiating PDAC from benign pancreatic tissue with a sensitivity of 92.5%, 80% and 72.5%, respectively and the specificity was of 100% for each marker. Among our control group, no one showed positive staining for any of the studied markers.

In the existing work, the expression of Maspin in PDAC cases was almost similar to the majority of studies in literature (>/= 90% of cases) and on the same line, all the benign pancreatic tissues were negative for it (Cao et al., 2007; Berardi et al., 2013; Aksoy-Altinboga et al., 2018). Aksoy-Altinboga et al., (2018) reported that using Maspin in the differential diagnosis of PDAC from benign/reactive pancreatic tissue achieved diagnostic sensitivity and accuracy of 87.5% and 91.2% respectively, which were slightly lower than what we recorded, while they recorded higher NPV of 89%, whereas specificity and PPV were similar to our findings (100%, each).

Regarding the subcellular localization of Maspin immunostaining positivity, there is wide variation in the literature with lack of standardization. For example, Cao et al., (2007) documented that 87% of cases had only cytoplasmic expression, while the majority of our Maspin positive PDAC cases (77%) had nuclear and cytoplasmic immunostaining. Berardi et al., (2013) and Banias et al., (2019) explained that the different position of Maspin inside the cell is related to epigenetic changes. They demonstrated that in malignant tumors, Maspin expression and its subcellular localization influence both tumor behavior and response to chemotherapy. However few studies in literature investigated the significance of subcellular localization of Maspin in PDAC. Cao et al., (2007) proved that nuclear labeling of Maspin in PDAC is associated with better tumor differentiation although it is not associated with a better prognosis. Berardi et al., (2013) showed that nuclear expression of Maspin in PDAC predicts a poor prognosis.

All studies of CK17 in PDAC come in literature were done on surgically resected specimens, with wide variation in positivity rate. Higher figures were noted by Chu et al., (2005), sarbia et al., (2007) and Yang et al., (2012), represented 83%, 88 % and 92% of PDAC cases, respectively. In contrast, CK17 was only found in 60 % of pancreaticobiliary adenocarcinomas (Goldstein and Bassi, 2001). Yang et al., (2012) also found that CK17 was totally negative in normal pancreas.

Few studies investigated the expression of Ki-67 in PDAC, with results reflect usage of variable cutoff points for positivity. Kim et al., (2015) found that Ki-67 was positive in 88.2% of the PDAC cases, considering 1 % as a cutoff point for positivity. Whereas a lower figure was reported by Jahng et al., (2010) who considered the intense immunostaining for greater than 50% of population as a cutoff point for positivity. They reported that 41% of PDAC cases were positive for Ki-67, while all benign cases of pancreatitis were negative.

In the present study, the majority of positive PDAC cases for Maspin and CK17 showed diffuse immunostaining and strong intensity. These results come in concordance with many studies (Chu et al., 2005; Cao et al., 2007; Liu et al., 2012; Aksoy-Altinboga et al., 2018). Whereas study conducted by Goldstein and bassi (2001) revealed that focal and diffuse immunostaining for CK17 are equally distributed among positive PDAC cases. Similarly, the majority of our positive PDAC cases for Ki-67 exhibited strong immunostaining intensity, while diffuse immunostaining only represented 22.41%. To the best of our knowledge, no studies in the literature studied the percentage of positive cells and intensity of Ki-67 immunostaining in PDAC.

In our study, for differentiation between PDAC and benign pancreatic tissue, we found that Maspin on its own had the highest sensitivity, NPV and accuracy, when compared with CK17 and Ki-67. Specificity and PPV were 100% for each marker. We demonstrated that combined use of Maspin, CK17 and Ki-67 as a triple panel with at least one of them is positive, achieved the highest sensitivity, NPV and accuracy of 98.8%, 96.2% and 99% for diagnosing PDAC, when compared to using each two of them separately (alternatively double panels).

Liu et al., (2012) studied 26 markers in the differentiation between PDAC and normal/non-neoplastic pancreatic tissues. In concordance with our study, all PDAC cases (100%) showed positive staining for Maspin. Whereas CK17 was only positive in 60 % of PDAC cases, which was much lower than we found. Both Maspin and CK17 were negative in all normal/non neoplastic pancreatic tissues. The same findings noticed by Lok et al., (2014) who evaluated immunohistochemical panel in distinction between intrahepatic cholangiocarcinoma and metastatic PDAC on a liver biopsy. CK17 was only seen in 60% of cases of PDAC, while all PDAC cases showed positive Maspin immunostaining.

Furhata et al., (2017) considered a higher cutoff point (26%) for Maspin positivity. They found that Maspin was positive in 40 % of PDAC and Ki-67 was positive in 53 % of PDAC, while no one of normal pancreatic ductal tissues was positive for both markers.

In correlation between our studied markers and studied clinico-pathological data, the only one was found to have statistical significant relation was Ki-67-positive tumors with tumor grade, compared with negative ones. Kim et al., (2015) similarly found that Ki-67 expression was correlated with pathological grade. They also found a correlation with lymph node, metastasis, and clinical stage. Whereas, Karamitopoulou et al., (2010) reported that Ki-67 labeling index did not show any signiﬁcant association with tumor grade, T stage or lymph node status. In contrast, both Maspin and CK17 immunostaining were not significantly associated with all our studied clinico-pathological data. Cao et al., (2007) also noticed that Maspin expression was not correlated with multiple clinico-pathologic variables. To the best of our knowledge, no studies in the literature correlated the significant relationship between CK17 immunostaining and clinico-pathological variables in PDAC.

To conclude, Maspin, CK17 and Ki-67 seem to be helpful markers in increasing the accuracy of diagnosing PDAC in EUS-FNAC. Usage of Maspin, CK17 and Ki-67 as a triple test (at least one of them is positive) is a potential ICC panel in the differentiation between PDAC and benign pancreatic tissue. Out of the 3 studied markers, the only one showed statistically significant relation with tumor grade is Ki-67 positive cases, compared with the Ki-67-negative ones.

## Author Contribution Statement

The authors confirm contribution to the paper as follows: study conception and design: Dr Mona M. Mamdouh, Dr Emad Hamza El-Gemeie, Dr Hussein Okasha, Dr HebatAllah M. Shaaban and Dr Nesreen H. Hafez.; data collection: Dr Mona M. Mamdouh; analysis and interpretation of results: Dr Mona M. Mamdouh, Dr Emad Hamza El-Gemeie, Dr Hussein Okasha, Dr HebatAllah M. Shaaban and Dr Nesreen H. Hafez.; draft manuscript preparation: Dr Mona M. Mamdouh. All authors reviewed the results and approved the final version of the manuscript.
